# 16S rRNA gene amplicon sequencing data from an Australian wastewater treatment plant

**DOI:** 10.1128/mra.01237-23

**Published:** 2024-05-03

**Authors:** C. S. Romanis, V. J. Timms, N. D. Crosbie, B. A. Neilan

**Affiliations:** 1 School of Environmental and Life Sciences, University of Newcastle, Callaghan, Australia; 2 ARC Centre of Excellence for Synthetic Biology, Newcastle, Australia; 3 Melbourne Water, Docklands, Victoria, Australia; California State University San Marcos, San Marcos, California, USA

**Keywords:** Cyanobacteria, wastewater treatment, wastewater, amplicon, QIIME

## Abstract

Amplicon sequencing data of the 16S rRNA (V1-V3) gene from 56 effluent and sediment samples from an Australian wastewater treatment plant are reported. Proteobacteria (3.50%–90.09%), Actinobacteria (0.02%–45.71%), and Cyanobacteria (0.05%–63.73%) were dominant in the effluent. The sediment samples were dominated by Proteobacteria (13.14%–84.83%), Chloroflexi (0.84%–42.52%), and Firmicutes (1.54%–17.21%).

## ANNOUNCEMENT

Wastewater treatment plants are hypereutrophic environments that utilize microorganisms for effluent remediation (1–3). Treatment efficacy is linked to the composition and stability of this microbial consortia (4–6). However, hypereutrophic conditions and long residence times can lead to seasonal proliferations of toxigenic cyanobacterial species (7–10), potentially altering nutrient availability and hindering remediation. The bacterial composition of the effluent at the Western Treatment Plant, Victoria (37.9310° S, 144.6370° E) was captured through 250 mL surface water grab samples collected fortnightly from 2018 to 2020 (Table 1). Quadruplicate surface water samples were collected and preserved on-site using Lugol’s iodine solution. Samples were filtered onto 0.22-µm glass microfiber filters and stored at −30°C (11, 12). Sediment cores were collected opportunistically using polyvinyl chloride pipe speared into the sludge to create a self-sealing plug. Genomic DNA from effluent and sediment samples (500 mg) was extracted using a published protocol (13). Sample and lysis beads were suspended in cetyltrimethyl ammonium bromide (CTAB) extraction buffer, 10% SDS, and N-lauroylsarcosine followed by extraction with phenol-chloroform-isoamyl alcohol (25:24:1) and overnight precipitation using 30% PEG 6000-1.6 M NaCl at 4°C. Extracted gDNA was stored at −30°C until processing. Amplification of the 16S V1-V3 rRNA gene was performed on ~25 ng gDNA using the KAPA HiFi HotStart ReadyMix (Roche) with the 27F (AGAGTTTGATCCTGGCTCAG) and 519R (GTATTACCGCGGCKGCTG) primers. Thermocycling conditions were denaturation (95°C for 3 min), 35 cycles of 98°C for 20 seconds, 55°C for 10 seconds, 72°C for 45 seconds, and extension (72°C for 5 min). Sequencing on an Illumina MiSeq using the MiSeq Reagent Kit v3 generated 300 bp paired-end reads.

**TABLE 1:** Characteristics and SRA accession numbers of the forward reads from sequences obtained from surface water and sediment samples of an Australian wastewater treatment plant

Sample ID	Sample type	Location in WSP^*a*^	Collection date	Raw reads	Processed reads	SRA accession
Sed12Ac	Sediment	Middle	25/02/2020	194,709	115,299	SRS18061223
Sed12Bc	Sediment	Middle	25/02/2020	390,317	208,833	SRS18061234
Sed12Cc	Sediment	Middle	25/02/2020	417,136	113,697	SRS18061245
Sed12D	Sediment	Middle	25/02/2020	116,476	97,394	SRS18061255
Sed13A	Sediment	Effluent	25/02/2020	131,521	103,249	SRS18061256
Sed13B	Sediment	Effluent	25/02/2020	159,005	124,987	SRS18061257
Sed13C	Sediment	Effluent	25/02/2020	71,518	54,197	SRS18061258
Sed13D	Sediment	Effluent	25/02/2020	194,917	113,738	SRS18061202
Sed3A	Sediment	Inlet	11/02/2020	196,539	161,815	SRS18061207
Sed3B	Sediment	Inlet	11/02/2020	333,741	293,658	SRS18061208
Sed3C	Sediment	Inlet	11/02/2020	336,074	281,403	SRS18061209
Sed3D	Sediment	Inlet	11/02/2020	567,205	459,350	SRS18061210
Sed8Ac	Sediment	Inlet	25/02/2020	182,975	43,378	SRS18061211
Sed8Bc	Sediment	Inlet	25/02/2020	17,375	9,327	SRS18061213
Sed8Cc	Sediment	Inlet	25/02/2020	122,218	90,495	SRS18061214
Sed8Dc	Sediment	Inlet	25/02/2020	150,738	79,804	SRS18061215
SedC1c	Sediment	Middle	11/02/2020	198,324	172,929	SRS18061216
SedC2c	Sediment	Middle	11/02/2020	210,156	152,316	SRS18061217
SedC3c	Sediment	Middle	11/02/2020	213,405	165,250	SRS18061218
SedC4c	Sediment	Middle	11/02/2020	574,468	219,365	SRS18061219
SedD2c	Sediment	Middle	11/02/2020	153,842	86,032	SRS18061220
SedD3c	Sediment	Middle	11/02/2020	316,170	168,212	SRS18061221
SedD4c	Sediment	Middle	11/02/2020	323,514	87,033	SRS18061222
SedE1	Sediment	NA	4/10/2018	137,336	114,438	SRS18061227
SedE3	Sediment	NA^*b*^	4/10/2018	76,576	58,202	SRS18061228
SedE4c	Sediment	NA	4/10/2018	127,075	69,104	SRS18061229
SedF1	Sediment	NA	31/07/2018	187,923	136,401	SRS18061233
SedF2	Sediment	NA	31/07/2018	85,533	69,660	SRS18061235
SedF3c	Sediment	NA	31/07/2018	173,867	147,362	SRS18061236
SedF4c	Sediment	NA	31/07/2018	69,349	55,106	SRS18061237
SedG1c	Sediment	NA	22/01/2019	69,354	45,675	SRS18061238
SedG2	Sediment	NA	22/01/2019	101,023	72,916	SRS18061239
SedG3	Sediment	NA	22/01/2019	196,255	166,966	SRS18061240
SedH1	Sediment	NA	13/02/2019	244,719	149,744	SRS18061241
SedH2	Sediment	NA	13/02/2019	232,433	174,367	SRS18061242
SedH4	Sediment	NA	13/02/2019	116,065	93,507	SRS18061244
Sed1T2	Water	Grab	4/10/2018	102,736	78,236	SRS18061203
Sed1T3	Water	Grab	4/10/2018	13,397	9,463	SRS18061204
Sed2T1	Water	Grab	31/07/2018	370,614	290,196	SRS18061205
Sed2T2	Water	Grab	4/10/2018	129,432	96,458	SRS18061206
SedD4	Water	Grab	29/01/2019	265,044	113,537	SRS18061224
SedD5	Water	Grab	29/01/2019	598,993	458,932	SRS18061225
SedD6	Water	Grab	29/01/2019	173,514	134,231	SRS18061226
SedE6	Water	Grab	11/02/2019	298,752	197,986	SRS18061230
SedE7	Water	Grab	11/02/2019	653,029	460,691	SRS18061231
SedE8	Water	Grab	11/02/2019	284,690	187,465	SRS18061232
SedS1T3	Water	Grab	31/07/2018	239,759	121,895	SRS18061243
SedS2T1	Water	Grab	31/07/2018	185,096	82,536	SRS18061246
SedS2T2	Water	Grab	31/07/2018	204,162	163,424	SRS18061247
WCE1	Water	Grab	10/02/2020	254,990	154,033	SRS18061248
WCE3	Water	Grab	10/02/2020	160,505	97,513	SRS18061249
WCE4	Water	Grab	10/02/2020	37,774	25,776	SRS18061250
WCF10	Water	Grab	25/02/2020	106,609	80,249	SRS18061251
WCF7c	Water	Grab	25/02/2020	198,088	131,892	SRS18061252
WCF8	Water	Grab	25/02/2020	152,093	102,540	SRS18061253
WCF9	Water	Grab	25/02/2020	191,266	133,578	SRS18061254

^*a*^ Waste stablisation pond (WSP).

^*b*^ "NA”-In circumstances where the precise sampling location within the WSP is not available, location information has been labelled as NA.

The Quantitative Insights Into Microbial Ecology (QIIME2) package (v2020.11) (14) was used to analyze forward sequence reads. QIIME2-DADA2 (15) was used for trimming 5 bp from the start of the raw reads and truncation to 240 bp to filter low-quality regions (PHRED score <34). Amplicon sequence variant (ASV) taxonomy was assigned using a naïve-Bayesian classifier generated within QIIME2, trained on the hypervariable V1-V3 16S rRNA gene of the SILVA reference database clustered at 99% sequence similarity (v138) (16). Sequence reads putatively identified as Cyanobacteria or chloroplast were aligned to the Cydrasil cyanobacterial sequence database (v3) (17), where any designations with a like weight ratio ≤0.4 were removed.

**Fig 1:**
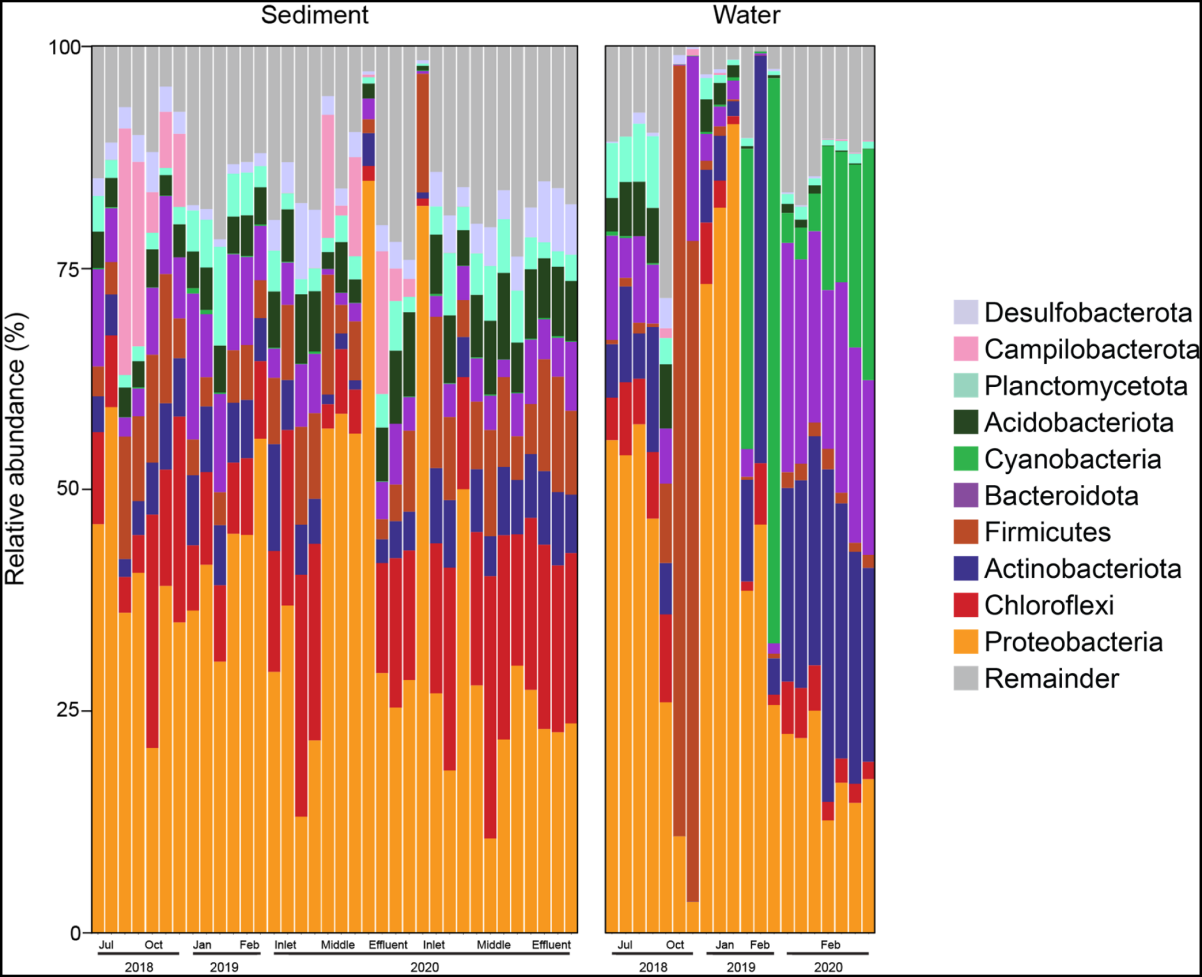
Relative abundance of the bacterial community composition of an Australian WWTP. ASVs were classified to the genus level using the SILVA database (v138). All 2020 sediment core samples were collected on the 11th and 25th of February from the pond inlet, mid-pond, or the pond outlet.

Sequencing of the 56 samples from the waste stabilization pond yielded 8,676,548 high-quality, non-chimeric sequence reads, from which 48,241 ASVs were identified. The relative abundance of sediment samples was dominated by Proteobacteria (13.14%–84.83%), Chloroflexi (0.84%–42.52%), and Firmicutes (1.54%–17.21%). The effluent was dominated by Proteobacteria (3.50%–90.09%), Actinobacteria (0.02%–45.71%), and Cyanobacteria (0.05%–63.73%) (Fig. 1). These findings, including biodiversity metrics, cyanobacteria migratory behavior, and *in vitro* validation are detailed in our related work (18). Surveillance of the wastewater treatment plants (WWTPs) bacterial composition empowers research of temporal trends in microbial composition and the effects of climate change on functionality and treatment efficiency.

## Data Availability

De-multiplexed sequencing data are on the NCBI Sequence Read Archive (SRA) under PRJNA987429. The SRA accession numbers are in Table 1. Study scripts are available on GitHub; https://github.com/c-romanis/Benthic_migration_paper.git and intermediate files accessible at https://doi.org/10.5281/zenodo.10021717.
